# Knowledge bases and software support for variant interpretation in precision oncology

**DOI:** 10.1093/bib/bbab134

**Published:** 2021-05-10

**Authors:** Florian Borchert, Andreas Mock, Aurelie Tomczak, Jonas Hügel, Samer Alkarkoukly, Alexander Knurr, Anna-Lena Volckmar, Albrecht Stenzinger, Peter Schirmacher, Jürgen Debus, Dirk Jäger, Thomas Longerich, Stefan Fröhling, Roland Eils, Nina Bougatf, Ulrich Sax, Matthieu-P Schapranow

**Affiliations:** Digital Health Center, Hasso Plattner Institute (HPI), University of Potsdam, Prof.-Dr.-Helmert-Str. 2-3, 14482 Potsdam, Germany; Department of Translational Medical Oncology (TMO), National Center for Tumor Diseases (NCT) Heidelberg, German Cancer Research Center (DKFZ) Heidelberg, Im Neuenheimer Feld 460, 69120 Heidelberg, Germany; Department of Medical Oncology, National Center for Tumor Diseases (NCT) Heidelberg, Heidelberg University Hospital, Im Neuenheimer Feld 460, 69120 Heidelberg, Germany; Institute of Pathology Heidelberg, Heidelberg University Hospital, Im Neuenheimer Feld 224, 69120 Heidelberg, Germany; Liver Cancer Center Heidelberg, Heidelberg University Hospital, Im Neuenheimer Feld 460, 69120 Heidelberg, Germany; Department of Medical Informatics, University Medical Center Göttingen, Von-Siebold-Str. 3, 37099 Göttingen, Germany; Campus Institute Data Science, Göttingen, Germany; CECAD, Faculty of Medicine and University Hospital Cologne, University of Cologne, Joseph-Stelzmann-Straße 26, 50931 Cologne; Division of Medical Informatics for Translational Oncology, German Cancer Research Center (DKFZ) Heidelberg, Im Neuenheimer Feld 280, 69120 Heidelberg, Germany; Institute of Pathology Heidelberg, Heidelberg University Hospital, Im Neuenheimer Feld 224, 69120 Heidelberg, Germany; Institute of Pathology Heidelberg, Heidelberg University Hospital, Im Neuenheimer Feld 224, 69120 Heidelberg, Germany; Institute of Pathology Heidelberg, Heidelberg University Hospital, Im Neuenheimer Feld 224, 69120 Heidelberg, Germany; Liver Cancer Center Heidelberg, Heidelberg University Hospital, Im Neuenheimer Feld 460, 69120 Heidelberg, Germany; Department of Radiation Oncology, Heidelberg University Hospital, Im Neuenheimer Feld 400, 69120 Heidelberg, Germany; National Center for Tumor Diseases (NCT), Heidelberg University Hospital, Im Neuenheimer Feld 460, 69120 Heidelberg, Germany; Clinical Cooperation Unit Radiation Oncology, German Cancer Research Center (DKFZ) Heidelberg, Im Neuenheimer Feld 280, 69120 Heidelberg, Germany; Heidelberg Ion-Beam Therapy Center (HIT), Department of Radiation Oncology, Heidelberg University Hospital, Im Neuenheimer Feld 450, 69120 Heidelberg, Germany; Heidelberg Institute of Radiation Oncology (HIRO), Heidelberg University Hospital, Im Neuenheimer Feld 400, 69120 Heidelberg, Germany; Department of Medical Oncology, National Center for Tumor Diseases (NCT) Heidelberg, Heidelberg University Hospital, Im Neuenheimer Feld 460, 69120 Heidelberg, Germany; Clinical Coorporation Unit Applied Tumor-Immunity, German Cancer Research Center (DKFZ) Heidelberg, Im Neuenheimer Feld 280, 69120 Heidelberg, Germany; Institute of Pathology Heidelberg, Heidelberg University Hospital, Im Neuenheimer Feld 224, 69120 Heidelberg, Germany; Liver Cancer Center Heidelberg, Heidelberg University Hospital, Im Neuenheimer Feld 460, 69120 Heidelberg, Germany; Department of Translational Medical Oncology (TMO), National Center for Tumor Diseases (NCT) Heidelberg, German Cancer Research Center (DKFZ) Heidelberg, Im Neuenheimer Feld 460, 69120 Heidelberg, Germany; German Cancer Consortium (DKTK), 69120 Heidelberg, Germany; Health Data Science Unit, Heidelberg University Hospital, Im Neuenheimer Feld 267, 69120 Heidelberg, Germany; Center for Digital Health, Berlin Institute of Health and Charité Universitötsmedizin Berlin, Kapelle-Ufer 2, 10117 Berlin, Germany; Department of Radiation Oncology, Heidelberg University Hospital, Im Neuenheimer Feld 400, 69120 Heidelberg, Germany; National Center for Tumor Diseases (NCT), Heidelberg University Hospital, Im Neuenheimer Feld 460, 69120 Heidelberg, Germany; Clinical Cooperation Unit Radiation Oncology, German Cancer Research Center (DKFZ) Heidelberg, Im Neuenheimer Feld 280, 69120 Heidelberg, Germany; Heidelberg Ion-Beam Therapy Center (HIT), Department of Radiation Oncology, Heidelberg University Hospital, Im Neuenheimer Feld 450, 69120 Heidelberg, Germany; Heidelberg Institute of Radiation Oncology (HIRO), Heidelberg University Hospital, Im Neuenheimer Feld 400, 69120 Heidelberg, Germany; Department of Medical Informatics, University Medical Center Göttingen, Von-Siebold-Str. 3, 37099 Göttingen, Germany; Campus Institute Data Science, Göttingen, Germany; Digital Health Center, Hasso Plattner Institute (HPI), University of Potsdam, Prof.-Dr.-Helmert-Str. 2-3, 14482 Potsdam, Germany

**Keywords:** HiGHmed, personalized medicine, molecular tumor board, data integration, cancer therapy

## Abstract

Precision oncology is a rapidly evolving interdisciplinary medical specialty. Comprehensive cancer panels are becoming increasingly available at pathology departments worldwide, creating the urgent need for scalable cancer variant annotation and molecularly informed treatment recommendations. A wealth of mainly academia-driven knowledge bases calls for software tools supporting the multi-step diagnostic process. We derive a comprehensive list of knowledge bases relevant for variant interpretation by a review of existing literature followed by a survey among medical experts from university hospitals in Germany. In addition, we review cancer variant interpretation tools, which integrate multiple knowledge bases. We categorize the knowledge bases along the diagnostic process in precision oncology and analyze programmatic access options as well as the integration of knowledge bases into software tools. The most commonly used knowledge bases provide good programmatic access options and have been integrated into a range of software tools. For the wider set of knowledge bases, access options vary across different parts of the diagnostic process. Programmatic access is limited for information regarding clinical classifications of variants and for therapy recommendations. The main issue for databases used for biological classification of pathogenic variants and pathway context information is the lack of standardized interfaces. There is no single cancer variant interpretation tool that integrates all identified knowledge bases. Specialized tools are available and need to be further developed for different steps in the diagnostic process.

## Introduction

The availability of molecular diagnostics for routine healthcare is democratizing the field of precision oncology, enabling molecularly informed treatment and clinical trials for a steadily increasing number of patients worldwide [1–6]. In parallel, molecular tumor boards (MTBs) are established in a growing number of hospitals to interpret the therapeutic consequences of molecular alterations [7–11]. The MTB is highly interdisciplinary and its composition may vary by hospital. Participating disciplines include among others: oncologists, pathologists, geneticists, bioinformaticians as well as other scientists and physicians from other medical specialties involved in the treatment of the patient, e.g. surgeons, gynecologists and neurologists, depending on the tumor entity. This review focuses on the interpretation of variants based on tumor sequencing without a concurrent germline sequencing. Nevertheless, human geneticists have been added to the team, as they help to decide on the necessity for consecutive germline testing. While sequencing capacities are scalable, variant annotation and prioritization remain the bottleneck in the diagnostic process and are not harmonized across cancer centers [12]. The American Society of Clinical Oncology (ASCO) Omics and Precision Oncology Workshop identified the lack of a single comprehensive Knowledge Base (KB) and the need to search multiple, sometimes conflicting resources as major challenges for applying omics in healthcare [13]. High-quality sequencing data and reusable data processing pipelines for the annotation and interpretation of sequencing data are emerging as the pillars of MTBs.

A number of software tools have recently been proposed to support the diagnostic workflow in precision oncology by providing unified interfaces to selected KBs [14–41]. In the remainder of our work, we will refer to this class of software as cancer variant interpretation tools. These tools take as an input either a list of variants, e.g. provided by a VCF file, or expose a query-oriented interface and return aggregated information retrieved from individual KBs with varying levels of integration and harmonization. This information can then be used to filter and prioritize variants and ultimately derive treatment suggestions. In order to be integrated into any software tool, KBs need to provide programmatic access options, e.g. through a public application programming interface (API) or by providing a dump of the data for downloading.

Even if the aforementioned access options are available, integration of heterogeneous data sources into software solutions is tedious and error prone, e.g. due to varying programmatic interfaces, data models and formats. Therefore, interoperability of data sources, data annotation and algorithms are key success factors of collaborative research and collaborative therapy finding. The German Federal Ministry of Education and Research also identified these challenges. Therefore, it provides funding for four national consortia in the German Medical Informatics Initiative through 2022 to develop national exchange strategies for data beyond individual university hospitals [42]. The given work was developed in the context of the HiGHmed consortium, one of the four funded consortia in this funding program [43]. Similar activities are also taking place in other European countries [44, 45].

Within the consortium, medical experts share their real-world observations and provide valuable feedback from their daily routine. As a result, the requirements and feedback described in this work represent the opinion of clinical experts working in university hospitals across Germany. Beyond the data sources, the workflows from biomaterial sampling and sequencing (panel, whole genome, or exome) to variant annotation and interpretation of the results vary widely from institution to institution. This represents a major challenge for the harmonization of the analysis pipelines in the medical use cases of the HiGHmed consortium and comparable use cases in other projects [46]. On an international level, the Global Alliance for Genomics and Health (GA4GH) aims to set technical standards and has launched the Variant Interpretation for Cancer Consortium (VICC) as a driver project.

### 1.1 Contributions

In this database review, we provide a systematic overview and categorization of KBs relevant for variant interpretation in oncology, with a particular focus on the technical challenges of their integration into software tools. We propose a categorization of KBs along the diagnostic process of precision oncology and will therefore begin our survey with a brief description of this process and the respective need to access KBs during each of its steps. For all KBs within their assigned categories, we gather general technical and category-specific non-technical parameters as well as bibliographic data on literature citations. In order to prioritize KBs by their relevance in clinical practice, we additionally present results of a survey among molecular pathologists and translational oncologists at university hospitals in the HiGHmed consortium. Based on this categorization, relevancy ranking and KB parameters, we identify engineering challenges for developers of cancer variant interpretation tools and directions for future research.

## 2 Background: diagnostic process

**Figure 1:**
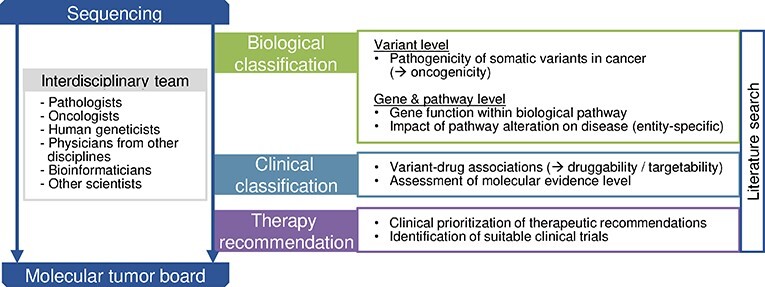
Overview of the diagnostic process in precision oncology. Within this process, knowledge bases can be attributed to the three main diagnostic steps biological classification of a variant, clinical classification and knowledge bases supporting therapy recommendations. Semi-automatic or manual literature search supports the entire process. The diagnostic process is performed by an interdisciplinary team of physicians and scientists. The exact composition and responsibilities within the team vary between university centers.

The diagnostic process in precision oncology from genome sequencing to the discussion of potential therapeutic consequences of molecular alterations requires both a biological and a clinical classification of variants (Figure 1). The process is highly interdisciplinary and physicians are commonly supported by both life and data scientists. Since precision oncology is a new and evolving discipline, the composition and responsibilities vary between university centers.

After obtaining the variant call format (VCF) file from the genetic data processing pipeline, biological classification of findings follows as the first diagnostic step. The term pathogenicity has been lend from the field of human genetics and its assessment includes population frequency, functional data, computational predictions, segregation and somatic frequency [47, 48]. The VICC suggests to use the term oncogenicity for the pathogenicity of somatic variants in cancer [49], but the nomenclature in the context of somatic analyses remains ambiguous.

Pathogenic variants are next interpreted in their pathway context and KBs are available that offer general information about the gene function within biological pathways and tissue- and tumor entity-specific expression patterns. However, most evidence about entity-specific pathway dependencies of variants has to be obtained by manual or semi-automated literature search, e.g. differences in oncogenic signaling between BRAF V600E-mutant melanomas and colorectal cancer.

The second step involves the clinical classification of variants, which is the identification of variant–drug or gene–drug associations, often referred to as druggability or targetability of a variant. In addition, this step includes the assessment of the molecular evidence level that is primarily defined by the clinical evidence of a study (prospective trial versus retrospective trial versus case study) as well as whether the study was performed within the same or a different tumor entity. In Germany, the National Center for Tumor Diseases (NCT) Heidelberg variant classification system is widely adapted. It provides evidence levels, which are of particular importance for the reimbursement of off-label molecularly informed drugs by German healthcare insurances. Leichsenring and colleagues present a comparison between the NCT and internationally recognized evidence levels [50].

The diagnostic process is, if available, concluded by therapy recommendations that are prioritized as the basis for discussion in the MTBs and for the search for suitable clinical trials.

## 3 Related work

In an early summary of the utility of NGS for cancer therapy, Gagan and Van Allen [51] recommend a set of 8 KBs to be used for the interpretation of somatic cancer variants. Tsang *et al.* [52] give an overview over catalogs of germline and somatic variants, functional annotation resources and resources linking cancer variants and clinical actionability. In addition, they describe software tools for manipulating variant datasets. Similarly, Prawira *et al.* [53] reviewed germline and somatic variant databases and *in silico* prediction tools. In contrast to the latter two reviews, we do not focus on effect predictions, unless they are part of a pre-computed databases such as dbNSFP [54]. Prediction of patient-individual effects describes another set of challenges beyond the scope of this study.

Zhang *et al.* [55] conducted an extensive review of computational resources (including databases, analysis tools and web platforms) for associations between diseases, genotypes, phenotypes and exposures. While the large set of KBs discussed in this work and ours overlap, we focus specifically on databases relevant for precision oncology with the goal to arrive at actionable treatment suggestions. Pallarz *et al.* [56] performed a qualitative and quantitative comparison of KBs for precision oncology and assessed their relative importance, concluding that each KB contains unique information relevant for MTB decisions.

In a recent, shorter review, Li and Warner [57] compiled a selection of publicly available and commercial KBs for determining therapeutic options for precision oncology, with detailed descriptions for each of the KBs. They also outline a high-level view of the sequencing process, whose steps ‘Interpretation’ and ‘Decision Making’ roughly correspond to the diagnostic process we described in Section 2. Also recently, Rao *et al.* [58] conducted a review of the landscape of tools and resources for the evaluation of cancer variants, including a survey of clinically relevant genomic data resources and KBs.

Our work aims to aggregate and extend the set of clinically relevant KBs for precision oncology discussed in the aforementioned articles, also including KBs of medical literature and registered clinical trials. In addition to existing comprehensive collections of KBs, we propose a categorization of KBs along the diagnostic process. Moreover, we attempt to guide implementers of cancer variant interpretation tools by an assessment of programmatic access options as well as a relevancy ranking based on feedback from clinical practitioners.

## 4 Methods

In the following, we share details about our involved methods to obtain the presented results.

### 4.1 Identification of relevant knowledge bases

First, we compiled a seed list of KBs based on prior reviews [52, 53, 57, 58] and guidelines for variant annotation by the medical societies ASCO [48] and ESMO [59]. This initial set contained 40 KBs (see the supplementary data). Second, we conducted a survey among a group of 10 selected medical professionals from hospital university centers in the HiGHmed consortium (university hospitals in Heidelberg, Göttingen, Cologne and the Hanover Medical School) and the German Cancer Research Center (DKFZ) to indicate which KBs are relevant for their daily work in the preparation of MTBs. This survey covered all involved parties in the recently implemented multi-site HiGHmed MTB. Participants were provided with the seed list and were asked to mark relevant and add missing KBs to the list. This way, 25 additional KBs were identified and added to the list, resulting in a total of 65 KBs. On the basis of our understanding of the critical difference between the biological and clinical variant interpretation introduced in Section 2, five participants of the survey were selected for their familiarity with biological variant interpretation (four molecular pathologists and one human geneticist), whereas the other five participants were selected from translational oncology departments (four oncologists and one bioinformatician from the German Cancer Research Center). The seed list of KBs and the survey responses were combined to assemble a complete list of KBs. The latter was sent to the participants once again, who had the opportunity to update their votes. The response options of the survey were yes, no and sometimes, e.g. when a KB was only used for specific tumor types. To simplify result presentation, we counted sometimes as yes and thus mapped the responses to a binary voting scheme. The detailed responses can be found in the supplementary data.

### 4.2 Categorization of knowledge bases along the diagnostic process

The KBs were categorized according to the diagnostic steps as introduced in Figure 1:

- Biological classification
- Clinical classification
- Therapy recommendation

Within the biological classification category, KBs providing information on the variant level are differentiated from KBs that contain gene or pathway-level information. In addition, we have gathered sources for targeted literature research. The categorization of KBs along the diagnostic process was consented by the medical practitioners among the authors based on their experience in the preparation of MTB cases and presented to the survey participants. In case a KB offered content for more than one diagnostic step, it was assigned to the category most used by the survey participants.

### 4.3 Assessment of technical and non-technical KB parameters

For each of the KBs, an in-depth analysis regarding the license (academic, commercial), programmatic access options and update intervals was performed by the authors. For access options, we distinguish accessibility via API, i.e. the data reside with the database maintainer and a possibility to download a complete or partial dump of the dataset. We did not consider databases as accessible via APIs if the only possibility was scraping of web content, even if this was not discouraged (e.g. via a robot.txt) or inhibited (e.g. via rate limitation) by the database maintainer. Information regarding APIs and dump options could typically be found on the respective project websites. Complete details including links to APIs and file servers are included in the supplementary data. When there was a possibility to get a dump of the data, we also aimed to determine the update frequency of these dumps. When this was not stated on the project websites, we estimated the update intervals by checking the timestamps on the respective file servers (typically FTP).

In addition to these technical parameters, category-specific parameters were determined for each KB, e.g. the emphasis on somatic or germline variants for KBs used for biological classification on the variant level, or the availability of evidence tiers in clinical classification KBs.

For KBs with accompanying research articles, we determine the number of citations from Google Scholar and normalize the citations per year. Exact values can be found in the supplementary files. In case there are multiple articles, as commonly encountered for updated KB version, we report the value for the article that received most citations per year.

### 4.4 Identification of cancer variant interpretation tools

We identified cancer variant interpretation tools that access multiple KBs through a systematic review of literature indexed in PubMed. Details of the review process with the corresponding PRISMA statement can be found in the supplementary data. We require that tools included in this survey are accessible either through a public demo website or by enabling local installation. To determine which KBs are accessible through each tool, we checked the accompanying websites, research publications and, when available, the source code. A brief manual evaluation of tools providing an online demo was performed with a small set of known pathogenic variants to assess the core functionalities. As our focus is the coverage of accessible KBs, we did not perform an evaluation of the respective search results in terms of correctness, completeness or currency of the integrated KBs. Furthermore, we did only consider potential tools that provide an interface on the variant level, i.e. tools that work solely on the level of genes were excluded. In addition, tools were not considered that required a paid account (e.g. VarSome Clinical [60]), were no longer maintained (e.g. Oncotator [17]) or otherwise inaccessible for testing (e.g. the VMTB knowledge base described by Pishvaian *et al.* [61]). The assessment of covered KBs was performed during manuscript preparation and last checked on 14 December 2020.

## 5 Results

In this section, we share the results gathered by the literature- and survey-based collection of KBs and software tools for variant interpretation.

### 5.1 Cancer variant interpretation tools

As a result of the systematic literature search, 26 tools are included. Incorporating feedback from survey participants, three of these tools [16, 39, 40] were added manually to the result set. These citations were either not indexed by PubMed or the abstract was missing. Additionally, we include the file-based annotation tools ANNOVAR [14], SnpEff, or the professional version ClinEff respectively [15] and the Ensembl Variant Effect Predictor (VEP) [20]. These were used as common building blocks of many other identified tools, which then add additional oncology-specific functionality around them. Furthermore, we list GATK Funcotator [18], which has replaced the no-longer maintained tool Oncotator [17].

We present the identified tools in Table 1 and indicate whether they take as an input a user query (query based) or a file of variants, such as a VCF file (file based), as well as whether they create a local copy of the source data (materialized) or whether the source databases are queried on the fly (API based). In addition, we list the type of output that is produced, distinguishing file-based annotations, search result sets combined from different KBs or domain-specific reports, e.g. for use in MTB preparation. We also indicate if there is a documented update mechanism to keep the integrated KBs current, and if so, whether the update is provided as a bundled release or whether there are individual update routines per KB. If the update mechanism could not be determined from the publication or source code, we indicate this as unknown.

**Table 1:** Overview of identified cancer variant interpretation tools, sorted by publication date. We indicate the type of interface (file or query based) for data input as well as the type of output, the accessibility of a online demo or the source code and the type of data integration and the corresponding update mechanisms. Details can be found in the supplementary data. PAS is available as an iOS app; therefore, online demo is marked in brackets.

**Tool**	**Cit.**	**Year**	**User interface**	**Output**	**Online demo**	**Source code**	**Data integration**	**Automatic KB update**
ANNOVAR	[14]	2010	File based	Annotation			Materialized	Individual
SnpEff/ClinEff	[15]	2012	File based	Annotation		✓	Materialized	Bundled
AnalyzeGenomes	[16]	2014	File based/query based	Result set	✓		Materialized	Individual
GATK Funcotator	[17, 18]	2015	File based	Annotation		✓	Materialized	Bundled
MyVariant.info	[19]	2016	Query based	Result set	✓		Materialized	Individual
Ensemble Variant Effect Predictor (VEP)	[20]	2016	File based	Annotation	✓	✓	Materialized	Bundled
CanProVar	[21, 22]	2017	Query based	Result set	✓		Materialized	Unknown
PathOS	[23]	2017	File based	Report	✓	✓	Materialized	Bundled
Houston Methodist Variant Viewer (HMVV)	[24]	2017	File based	Report		✓	Materialized	Bundled
MTB-Report	[25]	2018	File based	Report		✓	Materialized	Unknown
Smart Cancer Navigator	[26]	2018	Query based	Report	✓	✓	API based	API based
Clinical and Genomic Information System (CGIS)	[27]	2018	File based	Report	✓		Materialized	Unknown
PanDrugs	[28]	2018	File based/ query based	Report	✓		Materialized	Unknown
PREDICT Variant Information System (VIS)	[29]	2018	Query based	Result set	✓		Materialized	Individual
Sequence Variant Identification and Annotation Platform (SeqVItA)	[30]	2018	File based	Annotation		✓	Materialized	Bundled
Precision Medicine Knowledgebase (PreMedKB)	[31]	2019	Query based	Report	✓		Materialized	Unknown
Pathogenicity of Mutation Analyzer (PathoMAN)	[32]	2019	File based/ query based	Result set	✓		Materialized	Unknown
Variant Interpretation for Cancer (VIC)	[33]	2019	File based	Annotation		✓	Materialized	Bundled
Translational Genomics expert (TGex)	[34]	2019	File based/ query based	Report	✓		Materialized	Unknown
PAS	[35]	2020	Query based	Result set	(✓)		Materialized	Individual
AML Variant Analyzer (AMLVaran)	[36]	2020	File based	Report	✓	✓	Materialized	Bundled
Open Custom Ranked Analysis of Variants Toolkit (OpenCRAVAT)	[37]	2020	File based	Annotation		✓	Materialized	Individual
VICC Meta-Knowledgebase (VICC MetaKB)	[38]	2020	Query based	Result set	✓	✓	Materialized	Individual
MIRACUM-Pipe	[39]	2020	File based	Report		✓	Materialized	Individual
Molecular Tumor Board (MTB) Portal	[40]	2020	File based/ query based	Report	✓		Materialized	Individual
VarStack	[41]	2020	Query based	Result set	✓		Materialized	Unknown

Especially in the case of tools with very active development, e.g. OpenCRAVAT, the coverage of KBs is likely to increase with future versions.

### 5.2 Knowledge bases and software support

**Figure 2:**
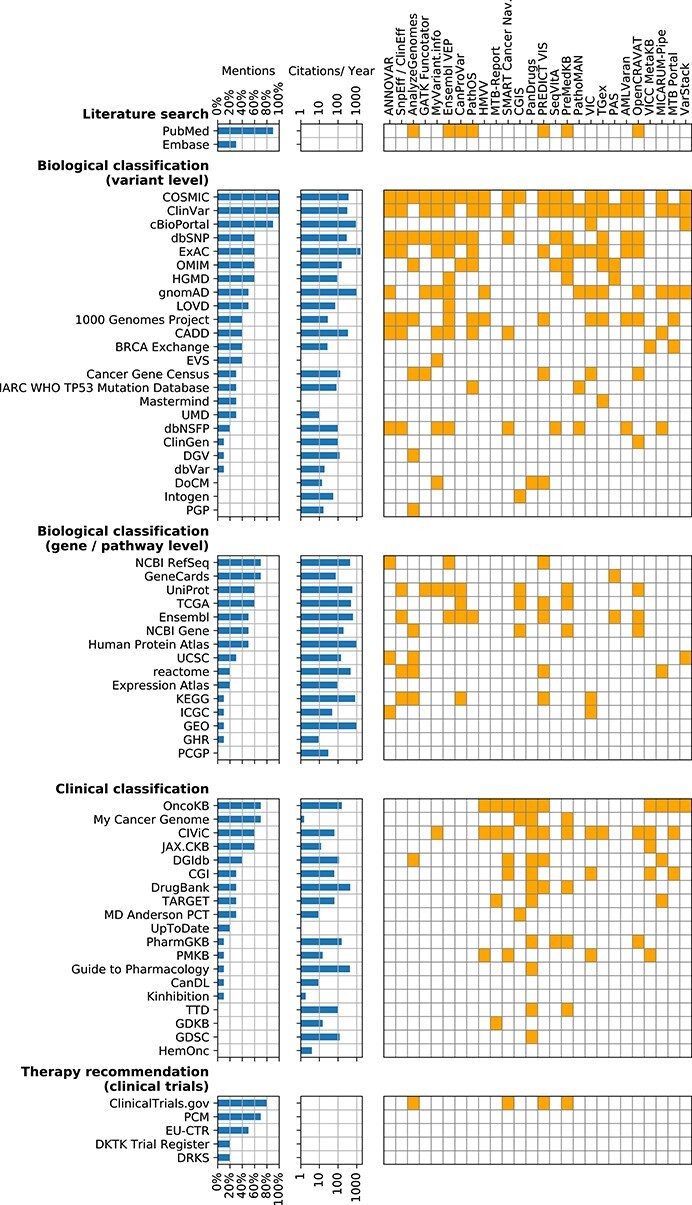
Survey results and tool integration for KBs. We report the overall fraction of survey participants who responded to use the KBs. The individual responses can be found in the supplementary data. We also report the number of citations per year according to Google Scholar. The right matrix indicates which KBs are accessible through each cancer variant interpretation tool.

In Tables 2–6, we list details on all identified KBs as well as their categorization along the diagnostic process. Figure 2 shows the ranking based on relevance in clinical practice indicated by subject-matter experts, as well as the coverage by the software tools introduced in the last section. We proceed by describing the main findings for each of the categories. For the first mention of a KB, we also report the percentage of participants responding in our survey to use the database in brackets.

**Table 2:** Literature search KBs. For each KB in this and the following tables, we indicate the proportion of survey mentions, the free availability for academic use as well as programmatic access options through APIs and/or downloading options of a dump. If the API or dump of a KB is not freely accessible, usually because of a commercial business model, this is indicated by parentheses around the checkmark. We also list the stated update interval of downloadable KB dumps or the estimated update interval (*) if details were not given on the respective project websites. No update interval (--) is reported, when a download is not available. For details, including URIs, see the supplementary data. As additional specific parameters for the literature KBs in this table, we report the number of included citations ($^\dagger $on 19 February 2021) as well as the controlled vocabulary used for indexing.

**Database**	**Source**	**Survey**	**Acad. use**	**API**	**Dump**	**Update interval**	**Number of citations** $^{\dagger }$	**Indexing**
PubMed	[63]	90%	✓	✓	✓	1d	>30M	MeSH
Embase	[64]	30%	–	(✓)	–	–	>32M	Emtree

Note that we do not report KBs, which are accessible through single tools but have not been identified through literature review or in the survey (see the Methods section). For instance, Molecular Match [62] is integrated into the VICC MetaKB but was not identified when compiling the list of KBs. Similarly, OpenCRAVAT integrates an ever increasing amount of KBs, many of which might not be of immediate relevance for clinical oncology and are therefore not discussed in this review.

We explicitly decide to report partially redundant KBs, if they have been named in the survey. For instance, prediction scores from the Combined Annotation Dependent Depletion (CADD) tool are available for downloading but also integrated into the Database of Human Nonsynonymous SNPs and Their Functional Predictions (dbNSFP). Similarly, The Exome Aggregation Consortium (ExAC) database has been completely migrated to the Genome Aggregation Database (gnomAD). In the case of partial or complete integration, this could reflect a preference of the survey participants to use the primary source of information. In the case of migration, it illustrates the challenge for tool developers to constantly update to the right version and location of each KB.

#### 5.2.1 Literature search

The identified KBs of scientific literature are shown in Table 2.

PubMed (90%) is, by a large margin, the most relevant literature search engine in our survey. The data behind PubMed (the MEDLINE and PubMed Central databases) and other NCBI resources can be downloaded in their entirety or via incremental daily updates from public FTP servers. In addition, the Entrez Programming Utilities (eUtils) can be used for programmatic access to various NCBI resources. This way, PubMed search can be readily integrated into software tools, even though advanced indexing beyond Medical Subject Headings (MeSH) and search functionalities relevant for variant annotation would have to be implemented on top of the PubMed data [65–68].

Embase (30%) as a commercial search engine is used by three survey participants, due to the accessibility of the latest conference abstracts and richer search functionalities compared to PubMed. Embase has an API that can be accessed once a license has been obtained.

Advanced or more specific literature search services such as Trip [69] or Livivo [70], or databases of systematic literature reviews such as the Cochrane Database of Systematic Reviews [71] were not mentioned by survey participants.

#### 5.2.2 Biological classification

In Tables 3 and 4, we show the KBs used for the biological classification on the level of variants, genes and pathways.

**Table 3:** Biological classification (variant level) KBs. In addition to the technical parameters and survey responses as introduced in Table 2, we report whether a KB contains information on somatic variants and/or germline variants as well as pre-computed functional prediction scores. We do not apply the somatic/germline distinction when databases provide functional prediction results only. Note that germline variants in cBioPortal are not publicly available. Further, note that in the included population/healthy controls data sets (ExAC, 1000 Genomes Project, EVS, DGV and PGP), somatic variants may be found occasionally; therefore, the column is checked in parentheses.

**Database**	**Source**	**Survey**	**Acad. use**	**API**	**Dump**	**Update interval**	**Somatic variants**	**Germline variants**	**Prediction scores**
COSMIC	[72]	100%	✓	✓	✓	3 m	✓		
ClinVar	[73]	100%	✓	✓	✓	1 w	✓	✓	
cBioPortal	[74]	90%	✓	✓	✓	1 w–1 m*	✓	(✓)	
dbSNP	[75]	60%	✓	✓	✓	Upon submission	✓	✓	
ExAC	[76]	60%	✓	–	✓	–	(✓)	✓	
OMIM	[77]	60%	✓	✓	✓	1 d	✓	✓	
HGMD	[78]	60%	✓	–	(✓)	3 m		✓	
gnomAD	[79]	50%	✓	–	✓	Last: October 2020	(✓)	✓	
LOVD	[80]	50%	✓	✓	✓	6 m	✓	✓	
1000 Genomes Project	[81]	40%	✓	✓	✓	Last: May 2013	(✓)	✓	
CADD	[82]	40%	✓	✓	✓	1 y	–	–	✓
BRCA Exchange	[83]	40%	✓	✓	✓	2 m		✓	
EVS	[84]	40%	✓	–	✓	Unknown	(✓)	✓	✓
Cancer Gene Census	[85]	30%	✓	–	✓	3 m	✓	✓	
IARC WHO TP53 Mutation Database	[86]	30%	✓	–	✓	1 y	✓	✓	✓
Mastermind	[87]	30%	(✓)	(✓)	–	–	✓	✓	
UMD	[88]	30%	✓	–	✓	Last: January 2013		✓	
dbNSFP	[54]	20%	✓	–	✓	3–6 m	–	–	✓
ClinGen	[89]	10%	✓	✓	–	–		✓	
DGV	[90]	10%	✓	–	✓	1–4 y	(✓)	✓	
dbVar	[91]	10%	✓	✓	✓	1–2 m	✓	✓	
DoCM	[92]	0%	✓	✓	–	–	✓		
Intogen	[93]	0%	✓	–	✓	Last: February 2020	✓		
PGP	[94]	0%	✓	–	✓	Upon submission	(✓)	✓	

**Table 4:** KBs for the biological classification on the gene/pathway level. In addition to the technical parameters and survey responses as introduced in Table 2, we indicate the available biological layers for each KB: genes (G), transcripts (T), proteins (P), gene expression (GE), protein expression (PE), pathways (PW) and other/multi-omics layers.

**Database**	**Source**	**Survey**	**Acad. use**	**API**	**Dump**	**Update interval**	**Biological layer**						
							**G**	**T**	**P**	**GE**	**PE**	**PW**	**Other**
NCBI RefSeq	[95]	70%	✓	✓	✓	1 d	✓	✓	✓				
GeneCards	[96]	70%	(✓)	(✓)	–	–	✓	✓	✓			✓	
UniProt	[97]	60%	✓	✓	✓	4 w			✓	✓	✓	✓	
TCGA	[98]	60%	✓	✓	✓	1–3 m	✓			✓	✓	✓	✓
Ensembl	[99]	50%	✓	✓	✓	3 m	✓	✓	✓	✓			
NCBI Gene	[100]	50%	✓	✓	✓	1 d	✓	✓	✓	✓		✓	
Human Protein Atlas	[101]	50%	✓	✓	✓	6–12m	✓		✓	✓	✓		
UCSC	[102]	30%	✓	✓	✓	2–8 w	✓			✓			✓
reactome	[103]	20%	✓	✓	✓	3–4 m	✓					✓	
Expression Atlas	[104]	20%	✓	✓	✓	2–5 m				✓			
KEGG	[105]	10%	✓	✓	✓	7 d	✓					✓	
ICGC	[106]	10%	✓	✓	✓	Last: March 2019	✓			✓	✓	✓	✓
GEO	[107]	10%	✓	✓	✓	Upon submission				✓			
GHR	[108]	10%	✓	✓	–	–	✓						
PCGP	[109]	0%	✓	–	(✓)	Unknown	✓			✓			✓

*Variant level:* databases in this category are mostly variant databases in structured tabular format. The most widely used KBs, ClinVar (100%) and the Catalogue Of Somatic Mutations In Cancer (COSMIC) (100%), are easily accessible through APIs and downloadable and have been integrated into a number of software tools. Opposed to that, cBioPortal (90%) is almost as widely used in practice and its data are accessible both via API and download, yet it has been integrated into only two of the considered tools.

Eight out of 17 databases most relevant for the survey participants (at least 30% mentions), namely cBioPortal, the Human Gene Mutation Database (HGMD) (60%), the Leiden Open Variation Database (LOVD) (50%), BRCA Exchange (40%), Exome Variant Server (EVS) (40%), the Universal Mutation Database (UMD) (30%), Mastermind (30%) and the International Agency for Research on Cancer (IARC) WHO TP53 Mutation Database (30%), have only been integrated into very few or no variant interpretation tools, even though programmatic access options exist. However, in the case of HGMD, the latest data are only available with a professional license and Mastermind provides a commercial API.

In contrast, the Database for Single Nucleotide Polymorphisms (dbSNP) (60%), Online Mendelian Inheritance in Man (OMIM) (60%), the 1000 Genomes Project (40%), the CADD (40%) database and Cancer Gene Census (30%) have been integrated into different tools. ExaC (60%) was also named as highly relevant, but has been migrated to gnomAD (50%) in the meantime, so the relative importance of gnomAD is expected to become larger in the future.

Less often used according to our survey, but publicly available and programmatically accessible resources are dbNSFP (20%), ClinGen (10%) and the Database of Genomic Variants (DGV) (10%), all which provide a download option or API access. The NCBI resource dbVar (10%) is mentioned in the ASCO guideline [48] and is regularly updated and programmatically accessible, yet it is not integrated into any considered tool.

While a large fraction of biological variant KBs are programmatically accessible and often integrated in a variety of tools, a more detailed investigation reveals that each one comes with their own individual data formats and interfaces. In effect, tools that access multiple of these databases have to implement separate ETL routines for each of the sources.

Three KBs (Personal Genomes Project (PGP) (0%), Database of Curated Mutations (DoCM) (0%) and Intogen (0%)) have been integrated into a few tools but are either unknown or irrelevant to our survey participants.

*Gene and pathway level:* the other category relevant for the biological classification of variants contains mainly KBs with information on the gene level. This includes general information about the gene function (NCBI RefSeq (70%), GeneCards (70%), Ensembl (50%), NCBI Gene (50%), the University of California Santa Cruz (UCSC) (30%) Genome Browser and Genetics Home Reference (GHR) (10%)), as well as insights about the tissue-specific (UniProt (60%), Human Protein Atlas (50%), Expression Atlas (20%), Gene Expression Omnibus (GEO) (10%)) or tumor entity-specific expression (The Cancer Genome Atlas (TCGA) (60%), International Cancer Genome Consortium (ICGC) (10%)).

A second type of KBs allow to query gene set level information in form of gene set enrichment analyses (reactome (20%), Kyoto Encyclopedia of Genes and Genomes (KEGG) (10%)).

An additional KB mentioned in the ASCO guideline on cancer variant interpretation [48], but not chosen in the survey, is the Pediatric Cancer Genome Project (PCGP) (0%).

Nearly all KBs in this category are programmatically accessible, yet none is integrated by more than a few tools, whereas in other categories, a set of a few important KBs are typically integrated by most tool providers.

#### 5.2.3 Clinical classification

In Table 5, we show the KBs used for the clinical classification step of the diagnostic process.

**Table 5:** Clinical classification KBs. In addition to the technical parameters and survey responses as introduced in Table 2, we report the availability of a system of evidence tiers, whether the curation process is based on manual curation by experts and/or a community, as well as the self-reported integration of text mining of scientific articles in the curation process.

**Database**	**Source**	**Survey**	**Acad. use**	**API**	**Dump**	**Update interval**	**Evidence tiers**	**Manual curation**	**Community**	**Text mining**
OncoKB	[110]	70%	✓	✓	✓	1–4 m*	✓	✓		
My Cancer Genome	[111]	70%	✓	(✓)	–	–		✓		
CIViC	[112]	60%	✓	✓	✓	1 m	✓	✓	✓	
JAX.CKB	[113]	60%	(✓)	–	(✓)	1 d	✓	✓		
DGIdb	[114]	40%	✓	✓	✓	1 m		✓		✓
CGI	[115]	30%	✓	✓	✓	Last: January 2018	✓	✓		
DrugBank	[116]	30%	✓	✓	✓	1–6 m*		✓		
TARGET	[117]	30%	✓	–	✓	Last: February 2015		✓		
MD Anderson PCT	[118]	30%	✓	–	–	–	✓	✓		✓
UpToDate	[119]	20%	–	(✓)	–	–		✓		
PharmGKB	[120]	10%	✓	✓	✓	Upon submission		✓		
PMKB	[121]	10%	✓	✓	✓	Last: August 2019	✓	✓		
Guide to Pharmacology	[122]	10%	✓	✓	✓	1-3 m		✓		
CanDL	[123]	10%	✓	–	✓	Last: July 2015	✓	✓		
Kinhibition	[124]	10%	✓	–	–	–				
TTD	[125]	0%	✓	–	✓	Last: June 2020	✓	✓		
GDKB	[126]	0%	✓	–	✓	Last: July 2017	✓	✓		
GDSC	[127]	0%	✓	–	✓	3 m–1 y*				
HemOnc	[128]	0%	✓	–	–	–	✓	✓	✓	

**Table 6:** Clinical trial registers and search engines. In addition to the technical parameters and survey responses as introduced in Table 2, we report the scope of trial locations, the number of registered trials ($^\dagger $on 19 February 2021) and the availability of an option to search by molecular markers in each KB.

**Database**	**Source**	**Survey**	**Acad. use**	**API**	**Dump**	**Update interval**	**Scope**	**Number of trials** $^{\dagger }$	**Molecular markers**
ClinicalTrials.gov	[129]	80%	✓	✓	✓	1 d	International	367 846	
PCM	[130]	70%	✓	–	–	–	International	314	✓
EU-CTR	[131]	50%	✓	–	–	–	EU	39 147	
DKTK Trial Register	[132]	20%	✓	–	(✓)	1 m	Germany	1056	✓
DRKS	[133]	20%	✓	–	–	–	Germany	11 354	

There are a number of widely used, programmatically accessible KBs with information on the clinical actionability of cancer variants. OncoKB (70%) and the community KB Clinical Interpretation of Variants in Cancer (CIViC) (60%) are accessible via API and dump options and are integrated into many recent annotation tools. Likewise, the The Drug Gene Interaction Database (DGIdb) (40%) provides similar programmatic access options and receives monthly updates since the major 4.x release. Cancer Genome Interpreter (CGI) (30%) provides a REST API and can be downloaded but has not been updated since 2018. Similarly, the data of Tumor Alterations Relevant for Genomics-Driven Therapy (TARGET) (30%) and Cancer Driver Log (CanDL) (10%) can still be downloaded but have not been updated since 2015. Somewhat less important resources, albeit still regularly updated, are DrugBank (30%), the Pharmacogenomics Knowledgebase (PharmGKB) (10%), the Precision Medicine Knowledge Base (PMKB) (10%) and Guide To Pharmacology (10%) with either API access and download options.

Commercial or otherwise programmatically inaccessible KBs play a comparatively important role when it comes to clinical variant classification. MyCancerGenome (70%) provides an API only on demand through its licensee GenomOncology. JAX.CKB (60%) has not been integrated into any tool besides the VICC MetaKB, as downloading of JAX.CKB requires a paid account. The web pages MD Anderson Personalized Cancer Therapy (PCT) (30%) and Kinhibition (10%) also do not provide any documented programmatic access options.

Similarly to variant-level biological classification, KBs with information on targeted therapies are usually available in a structured tabular format. However, for clinical classification, there have been harmonization efforts for a range of KBs through the VICC Consortium [38]. From this category, the VICC MetaKB incorporates OncoKB, CIViC, JAX.CKB, Cancer Genome Interpreter, PMKB and Molecular Match. Indeed, this seems to cover most of the identified clinical classification KBs that provide their data for downloading.

Synthesized evidence appears to play only a minor role in precision oncology. Commercial resources such as UpToDate (20%) have not been integrated into any tools under consideration. Other forms of evidence synthesis, such as clinical practice guidelines, also seem to be of little relevance in variant interpretation, as targeted therapies are mostly beyond the scope of guideline recommendations. Also, expectedly, resources on clinical classifications tend to be cancer specific (see the supplementary data).

The Gene Drug Knowledge Base (GDKB) (0%), Therapeutic Target Database (TTD) (0%) and Genomics of Drug Sensitivity in Cancer (GDSC) (0%) are accessible through single tools but were either unknown or irrelevant to our survey participants. The Wiki-based HemOnc (0%) has been named by prior reviews on the subject (e.g. Li and Warner [57]) but is not integrated into any of the tools nor was it mentioned by survey participants.

Even though evidence levels for gene–drug or variant–drug associations are not standardized across KBs, it is helpful if they include a form of evidence grading to guide identification of molecularly informed cancer treatments. KBs that provide some form of evidence tiers include OncoKB, CIViC, MD Anderson PCT, JAX.CKB, Cancer Genome Interpreter, Gene Drug Knowledge Base, PMKB, HemOnc, TTD and CanDL.

While most of the KBs in this category ultimately consist of curated evidence from the primary literature, only very few maintainers report to make use of text mining in the curation process.

#### 5.2.4 Therapy recommendation (clinical trials)

Finally, the KBs of clinical trials are shown in Table 6.

ClinicalTrials.gov (80%) is the most relevant database to search for clinical trials. It can be easily accessed via a REST API and downloaded in an XML format with daily updates. In effect, ClinicalTrials.gov has been integrated into a few different software tools and can be augmented with indices for variants and genes [134]. Precision Cancer Medicine (PCM) (70%) provides a specialized trials search engine to find trials focusing on targeted therapies. Among survey participants, it is perceived as almost as important as ClinicalTrials.gov but in contrast does not provide means for programmatic access and has therefore not been integrated into any of the software tools we consider. Even though the curation process of PCM is unclear, its widespread use emphasizes the demand for specialized search engines for biomarker driven clinical trials.

In addition to well-known international trial registers, finding matching local trials is highly relevant for actual treatment suggestions, as location will be an important factor for inclusion in ongoing trials. In effect, study registers have been mentioned to be relevant which are specific to Europe, such as the EU Clinical Trials Register (EU-CTR) (50%), or to Germany, such as the German Clinical Trials Register (DRKS) (20%) or the German Cancer Consortium (DKTK) Trial Register (20%), which integrates local trial registers from individual sites of the German Cancer Consortium. We expect additional trial registers to be relevant in other countries.

Except for ClinicalTrials.gov, programmatic accessibility to clinical trial data is very restricted. For instance, the EU-CTR does not provide an official API or download options, even though the plain text content of the EU-CTR website could potentially be scraped fairly easily [135].

### 5.3 Analysis of citations

The number of citations per year for each KB is displayed in Figure 2. As literature search engines and clinical trial registers are typically not explicitly referenced, the number of citations for these categories are 0. Spearman’s rank correlation coefficients between survey mentions, citations per year and number of tools that integrate a certain KB for each of the other three categories are given in Table 7.

There is a significant correlation between both the number of citations and mentions in the survey and the number of integrating tools for variant level biological classification KBs. For gene/pathway level KBs, there is no such correlation. For clinical classification KBs, the correlation with tool support is only significant for our survey results, not for citations in the literature. Again, this can most likely be attributed to the fact that some KBs in this category are less driven by academia.

**Table 7:** Correlation of citations and survey mentions of KBs with their availability through software tools per category. The left columns show the Spearman rank correlation between the number of citations per year and the number of tools integrating a certain KB. The right columns show the Spearman rank correlation between the number of survey mentions and the number of tools integrating a certain KB. Significant values ($P <.05$) are underlined.

	**Citations/no. tools**		**Survey/no. tools**	
	$\rho $	$P$	$\rho $	$P$
Biological classification (variant level)	.65	<.001	.66	<.001
Biological classification (gene/pathway level)	.38	.167	.40	.138
Clinical classification	.46	.057	.58	.011

### 5.4 Update intervals

The identified update intervals of downloadable resources range from immediate updates upon submission, regular updates in larger intervals (weekly, monthly, yearly) to irregular major releases in the range of months to years. Many databases integrated into some of the tools have not been updated in multiple years.

With few exceptions (e.g. some of the NCBI resources), most KB providers do not provide straightforward options to perform incremental updates. This poses a substantial challenge for the implementation of automated updating mechanisms in tools based on materialized integrated KBs, as the different update intervals of every source database need to be considered.

Our data show a correlation between documented dump options, update intervals and integration of KBs by software tools. As almost all tools are based on materialized representations, KBs without a dump option have been hardly integrated into any tools (see Tables 2–6 and Figure 2). For the subset of tools with verifiable update intervals ($n=43$), the length of the update interval (or the time since the last update) is significantly negatively correlated with both the proportion of mentions in the survey (Spearman rank correlation test $\rho =-.61$, $P<.001$) and the number of integrating tools ($\rho =-.37$, $P=.014$), i.e. KBs with frequent updates tend to be integrated by more tool developers.

## 6 Discussion

### 6.1 Tool support for variant interpretation

Molecular diagnostics in precision oncology is an inter-disciplinary process comprising several steps. The review of cancer variant interpretation tools revealed that not a single tool covers all KBs required for all steps equally. Depending on the diagnostic steps, the survey results suggest the usage of specific tools. In addition, the present work allows to determine the possibility to interrogate which tools and which KBs might be integrated in an individualized automated workflow.

We found that for earlier diagnostic steps, especially the biological classification on the variant level, there was a stronger consensus among tool developers about relevant KB to integrate. This consensus is reflected by the strong correlation between the number of integrating tools, citations and mentions in the survey. KBs containing information on the gene or pathway level were identified to be underrepresented in cancer variant interpretation tools and were mentioned by relatively fewer survey participants. While some of these KBs are highly cited in the scientific literature, the relevance according to these citations and also the mentions in our survey are not reflected by the number of integrating tools. However, this step might gain more importance in the future when clinical-grade exome sequencing and RNA-seq become available.

### 6.2 Standards and interoperability

Our investigation regarding programmatic accessibility reveals notable disparities among categories of KBs used along the diagnostic process. Ongoing standardization efforts within the VICC consortium of the GA4GH initiative address mostly KBs with information on targeted therapies. Defining such harmonized interfaces, based on syntactic and semantic standards, will also facilitate the integration of in-house databases within and across hospitals. However, the relative importance of commercial or otherwise programmatically inaccessible KBs will remain an obstacle for creating a comprehensive integrated KB.

Surprisingly, programmatic access to ongoing clinical trial information, even though in essence publicly available, is very restricted, with ClinicalTrials.gov being an exception. Opposed to that, while most important KBs used for biological classification provide programmatic access options, there is little harmonization and standardization across database providers, imposing a significant burden for the implementation and maintenance of cancer variant interpretation tools integrating these KBs.

### 6.3 Update intervals and mechanisms

Aligning software tool release cycles with KB releases is a major challenge to be addressed by tool providers. Vastly different update intervals and a lack of incremental updates for many KBs makes updating materialized representations on a per-KB basis challenging. Tools that still enable such updates have to implement KB specific extract-transform-load routines. In practice, users will also need to know if the currently used KB version is the latest version available. Managing these updates will be an additional burden to users.

Tools that provide bundled releases might be more convenient in this regard but add an additional layer of indirection that might incur additional time lags in translation of new results into clinical practice. Moreover, the exact update mechanism has been not clearly documented for a number of identified annotation tools, although this information will be essential for the integration into clinical workflows.

### 6.4 Usability

Integration of a large set of relevant KBs is a deciding factor, but not the only design aspect motivating the choice of a particular cancer variant interpretation tool. Software usability will be an important feature for adoption by clinical practitioners. For instance, a query-based interface requiring exact input of variant coordinates can impede an explorative use of a tool, in particular in later stages of the diagnostic process. Potentially large results sets from different KBs need to be presented in a manageable fashion, e.g. by providing filtering and sorting mechanisms with sensible defaults. In addition, a usable tool should provide adequate export functionalities to integrate well with applications downstream in the treatment process, e.g. tumor board reporting tools.

### 6.5 Implementation of an audit trail

A crucial consideration regarding the technical mechanisms used to integrate different KBs is the implementation of an audit trail. When data in public KBs is used to give treatment recommendations endorsed by an MTB, there needs to be a possibility to refer and keep track of the version of the data the decision was based on. Apart from data privacy and performance considerations, this is a key aspect motivating the use of materialized representations of the source data as opposed to an API-based integration. The application of automated versioning of these data in a database management system would be one natural way to implement such an audit trail. In addition to these technical parameters, metadata about the quality of the data sources, the curation process and the data integration pipelines are needed. Usually, these data are not provided in the standard interfaces.

This audit trail would include the information about the queried KBs, their version, the query result and the influence of the result on the MTB recommendation [136, 137]. Furthermore, findability, accessibility, interoperability and reusability of the provided data and pipelines are mandatory to allow implementation of the FAIR guiding principles for scientific data [138]. Most of the aspects are already considered in this review (see Tables 2–6), yet the undetermined update cycles of some data sources are problematic in terms of findability and reusability.

### 6.6 Annotation quality and redundancy

In previous studies, it has been established that subsets of the identified KBs contain partially overlapping information [26, 38, 56, 139]. Performing a similar analysis on the large set of KBs covered in this survey is likely to reveal similar partial or complete subsumption among KBs, as well as disagreement in annotations. Reliable identification of such conflicts is a prerequisite for developing strategies for their resolution [29]. While this information would be extremely valuable to implementers of cancer variant interpretation tools, such an analysis is hardly feasible without prior restructuring of all KBs to a canonical data format, as it was done in the aforementioned studies, and therefore remains an open problem.

### 6.7 Determination of molecular evidence levels

The principles of evidence-based medicine also apply to precision oncology. Different variant classification systems have been proposed assigning evidence to variant-drug associations in the context of the individual tumor entity [50]. Main principles of molecular evidence levels include a higher evidence for studies in the same tumor entity versus a different entity and for prospective studies versus retrospective studies or case reports. While several KBs (see Table 5) list evidence levels, they rely on manual curation and are far from being complete. As of today, clinical assertions in these KBs also cannot be systematically and automatically derived through, e.g. text mining of publications, even though this is an active area of research [140, 141]. In Germany, the molecular evidence level determines whether an off-label therapy qualifies for reimbursement by health insurances and is hence an essential part of the MTB report but not a single KB offers an annotation according the NCT evidence levels. Hence, the determination of molecular evidence levels remains a mainly manual and time-consuming step in precision oncology.

### 6.8 Limitations

#### 6.8.1 Selection of knowledge bases

While we based the selection of KBs on a variety of existing reviews, guidelines and a survey across multiple hospital sites in a large national consortium, a different choice of survey participants could have resulted in a deviating set of KBs. This would likely be the case in particular for KBs, which were included because they were named by a single survey participant. However, as there was substantial agreement regarding the most important KBs, we expect the overall ranking to be informative. In addition, we expect further national databases to be important in other countries, for instance when it comes to finding matching local clinical trials. As the field of precision oncology is evolving at a rapid pace, the list of KBs, their relative importance and coverage by tools are bound to be a temporal snapshot of the current state-of-the-art in variant interpretation.

#### 6.8.2 Variability across institutions

A number of issues regarding the integration of KBs into the diagnostic process have not been considered in this survey. In particular, different sequencing technologies and variant calling pipelines are used across university centers that may or may not already include variant filters. The impact of this variability on the relevance of used KBs as indicated in the survey needs to be considered when interpreting our results. A better understanding of the differences between variant calling pipelines will be crucial to harmonize variant annotation and prioritization workflows in the future.

### 6.9 Relevance of commercial solutions

In this work, we deliberately chose to only include non-commercial cancer variant interpretation tools to support open science and to enable a unified software support within multiple academic medical centers in Germany independent of individual licenses. Alongside these ongoing efforts at university hospitals, several commercial software solutions are widely used at hospitals and private practices in Germany including the NAVIFY Mutation Profiler (Roche), QIAGEN Clinical Insight (QCI) Interpreter, CureMatch Bionov, Molecular Health Guide and Sophia Genetics. Common advantages include user friendly front ends, end-to-end workflows from sequencing files to reports and audit trails. However, a direct comparison of the KB integration of academic and commercial is not feasible since the exact use and weighting of data sources is in most cases protected and the closed source architecture impedes an integration with other KBs deemed important for the individual tumor of study.

## 7 Conclusion and outlook

The landscape of KBs for variant annotation and interpretation in precision oncology is constantly evolving. In this database review, our goal was to give an up-to-date overview of the most important precision oncology KBs relevant for molecular pathologists and translational oncologists. In addition, we discussed programmatic access options of KBs and their integration into cancer variant interpretation tools. While it only shows a point-in-time record and may not reflect a European or international opinion, it still provides major building blocks for them.

When adopting any of the KBs in diagnostic workflows, attention must be given to the curation process and the resulting data quality. This is an urgent matter as comprehensive gene panel analysis is becoming a routine diagnostics method at an increasing number of academic cancer centers worldwide. Therefore, completeness and currency of the results derived from interpretation tools does not only depend on automated and errorless data integration mechanisms but also on the reliability of the underlying data sources. A quantitative evaluation of different cancer variant interpretation tools in this regard will be an important direction for future research.

A major future challenge for software tools supporting the diagnostic process in precision oncology is the integration of additional biological layers, particularly the transcriptome and methylome. RNA sequencing has been pivotal to reliably detect cancer gene fusions [142] and help guide cancer of unknown primary diagnostics [143–146]. In addition, an RNA overexpression of candidate driver genes could be identified as predictive biomarkers in the absence of genetic alterations in the same gene [147, 148]. The methylome is gaining increasing significance for diagnostic purposes, e.g. in the classification of central nervous tissue tumors [149] and cancers of unknown primary [150, 151]. Another emerging layer that could aid to increase the performance of drug response predictions is proteomics [152–154].

Lastly, as more and more patients receive targeted treatment based on molecular diagnostics, a key question for future projects will be how outcome data of these cases can be obtained, shared and used to inform evidence-based decision making. Community KBs are a step in this direction, but maintenance and sharing of in-house databases within and across hospitals will become pressing issues in the near future.

**Key Points**

$\bullet $ Variant interpretation in precision oncology requires access to a variety of knowledge bases in different parts of the diagnostic process.

$\bullet $ Through a review of literature and guidelines for variant interpretation as well as a survey among clinical practitioners, we derive a comprehensive list of knowledge bases with a categorization along the diagnostic process.

$\bullet $ We assess programmatic access options for all identified knowledge bases and existing integrations into cancer variant interpretation tools .

## Supplementary Material

Suppl1_Cancer_Variant_Interpretation_Tools_bbab134

Suppl2_Knowledge_Bases_bbab134

Suppl3_Tool_Literature_Screening_bbab134

Suppl4_Prisma_Flow_Diagram_bbab134

## Notes

**Florian Borchert** is a computer scientist and PhD student at the Digital Health Center of the Hasso Plattner Institute (HPI) for Digital Engineering in Potsdam.

**Andreas Mock** is a physician–scientist at the National Center for Tumor Diseases (NCT) Heidelberg, German Cancer Research Center (DKFZ) and Heidelberg University Hospital.

**Aurelie Tomczak** is a bioinformatician at the Institute of Pathology Heidelberg, Heidelberg University Hospital and manages the patient register of the Liver Cancer Center Heidelberg.

**Jonas Hügel** is a computer scientist and PhD student in the Translational Research Informatics research group at the Department of Medical Informatics at the University Medical Center Göttingen (UMG).

**Mohammad Samer Alkarkoukly** is a physician and currently working as a medical data steward at the University Hospital Cologne for the HiGHmed project.

**Alexander Knurr** is a computer scientist working for more than 10 years at the NCT Heidelberg and the DKFZ.

**Anna-Lena Volckmar** is a biologist and performs routine diagnostic genetic testing at the Center for Molecular Pathology at the Institute of Pathology, Heidelberg University Hospital.

**Albrecht Stenzinger** heads the Center for Molecular Pathology at the Institute of Pathology, Heidelberg University Hospital.

**Peter Schirmacher** is the medical director of the Department of General Pathology and Pathological Anatomy and the managing director of the Institute of Pathology at Heidelberg University Hospital.

**Jürgen Debus** is the medical director of the Department of Radiation Oncology and a managing director of the NCT Heidelberg at Heidelberg University Hospital.

**Dirk Jäger** is the medical director of the Department of Medical Oncology at Heidelberg University Hospital, managing director of the NCT Heidelberg and head of the Clinical Cooperation Unit Applied Tumor-Immunity.

**Thomas Longerich** is the Deputy Medical Director of the Department of General Pathology and Pathological Anatomy at the Institute of Pathology, Heidelberg University Hospital.

**Stefan Fröhling** is a managing director of the NCT Heidelberg and head of the Department of Translational Medical Oncology at NCT Heidelberg and the DKFZ.

**Roland Eils** is the founding director of the Center for Digital Health at Berlin Institute of Health (Charité, Berlin) and the Health Data Science unit at the Medical Faculty of Heidelberg University.

**Nina Bougatf** is a computer scientist with high expertise in oncology and heads the NCT Cancer Registry and a data science research group at the Department of Radiation Oncology.

**Ulrich Sax** is a medical informaticist heading the Translational Research Informatics research group in the department of Medical Informatics at UMG.

**Matthieu-P. Schapranow** is Scientific Manager Digital Health Innovations at the HPI and head of the working group ‘In-Memory Computing for Digital Health’ at the Digital Health Center.
