# Knowledge bases and software support for variant interpretation in precision oncology

**DOI:** 10.1093/bib/bbab246

**Published:** 2021-06-14

**Authors:** Florian Borchert, Andreas Mock, Aurelie Tomczak, Jonas Hügel, Samer Alkarkoukly, Alexander Knurr, Anna-Lena Volckmar, Albrecht Stenzinger, Peter Schirmacher, Jürgen Debus, Dirk Jäger, Thomas Longerich, Stefan Fröhling, Roland Eils, Nina Bougatf, Ulrich Sax, Matthieu-P Schapranow

**Affiliations:** Digital Health Center, Hasso Plattner Institute (HPI), University of Potsdam, Prof.-Dr.-Helmert-Str. 2-3, 14482 Potsdam, Germany; Department of Translational Medical Oncology (TMO), National Center for Tumor Diseases (NCT) Heidelberg, German Cancer Research Center (DKFZ) Heidelberg, Im Neuenheimer Feld 460, 69120 Heidelberg, Germany; Department of Medical Oncology, National Center for Tumor Diseases (NCT) Heidelberg, Heidelberg University Hospital, Im Neuenheimer Feld 460, 69120 Heidelberg, Germany; Institute of Pathology Heidelberg, Heidelberg University Hospital, Im Neuenheimer Feld 224, 69120 Heidelberg, Germany; Liver Cancer Center Heidelberg, Heidelberg University Hospital, Im Neuenheimer Feld 460, 69120 Heidelberg, Germany; Department of Medical Informatics, University Medical Center Göttingen, Von-Siebold-Str. 3, 37099 Göttingen, Germany; Campus Institute Data Science, Göttingen, Germany; CECAD, Faculty of Medicine and University Hospital Cologne, University of Cologne, Joseph-Stelzmann-Straße 26, 50931 Cologne; Division of Medical Informatics for Translational Oncology, German Cancer Research Center (DKFZ) Heidelberg, Im Neuenheimer Feld 280, 69120 Heidelberg, Germany; Institute of Pathology Heidelberg, Heidelberg University Hospital, Im Neuenheimer Feld 224, 69120 Heidelberg, Germany; Institute of Pathology Heidelberg, Heidelberg University Hospital, Im Neuenheimer Feld 224, 69120 Heidelberg, Germany; Institute of Pathology Heidelberg, Heidelberg University Hospital, Im Neuenheimer Feld 224, 69120 Heidelberg, Germany; Liver Cancer Center Heidelberg, Heidelberg University Hospital, Im Neuenheimer Feld 460, 69120 Heidelberg, Germany; Department of Radiation Oncology, Heidelberg University Hospital, Im Neuenheimer Feld 400, 69120 Heidelberg, Germany; National Center for Tumor Diseases (NCT), Heidelberg University Hospital, Im Neuenheimer Feld 460, 69120 Heidelberg, Germany; Clinical Cooperation Unit Radiation Oncology, German Cancer Research Center (DKFZ) Heidelberg, Im Neuenheimer Feld 280, 69120 Heidelberg, Germany; Heidelberg Ion-Beam Therapy Center (HIT), Department of Radiation Oncology, Heidelberg University Hospital, Im Neuenheimer Feld 450, 69120 Heidelberg, Germany; Heidelberg Institute of Radiation Oncology (HIRO), Heidelberg University Hospital, Im Neuenheimer Feld 400, 69120 Heidelberg, Germany; Department of Medical Oncology, National Center for Tumor Diseases (NCT) Heidelberg, Heidelberg University Hospital, Im Neuenheimer Feld 460, 69120 Heidelberg, Germany; Clinical Coorporation Unit Applied Tumor-Immunity, German Cancer Research Center (DKFZ) Heidelberg, Im Neuenheimer Feld 280, 69120 Heidelberg, Germany; Institute of Pathology Heidelberg, Heidelberg University Hospital, Im Neuenheimer Feld 224, 69120 Heidelberg, Germany; Liver Cancer Center Heidelberg, Heidelberg University Hospital, Im Neuenheimer Feld 460, 69120 Heidelberg, Germany; Department of Translational Medical Oncology (TMO), National Center for Tumor Diseases (NCT) Heidelberg, German Cancer Research Center (DKFZ) Heidelberg, Im Neuenheimer Feld 460, 69120 Heidelberg, Germany; German Cancer Consortium (DKTK), 69120 Heidelberg, Germany; Health Data Science Unit, Heidelberg University Hospital, Im Neuenheimer Feld 267, 69120 Heidelberg, Germany; Center for Digital Health, Berlin Institute of Health and Charité Universitötsmedizin Berlin, Kapelle-Ufer 2, 10117 Berlin, Germany; Department of Radiation Oncology, Heidelberg University Hospital, Im Neuenheimer Feld 400, 69120 Heidelberg, Germany; National Center for Tumor Diseases (NCT), Heidelberg University Hospital, Im Neuenheimer Feld 460, 69120 Heidelberg, Germany; Clinical Cooperation Unit Radiation Oncology, German Cancer Research Center (DKFZ) Heidelberg, Im Neuenheimer Feld 280, 69120 Heidelberg, Germany; Heidelberg Ion-Beam Therapy Center (HIT), Department of Radiation Oncology, Heidelberg University Hospital, Im Neuenheimer Feld 450, 69120 Heidelberg, Germany; Heidelberg Institute of Radiation Oncology (HIRO), Heidelberg University Hospital, Im Neuenheimer Feld 400, 69120 Heidelberg, Germany; Department of Medical Informatics, University Medical Center Göttingen, Von-Siebold-Str. 3, 37099 Göttingen, Germany; Campus Institute Data Science, Göttingen, Germany; Digital Health Center, Hasso Plattner Institute (HPI), University of Potsdam, Prof.-Dr.-Helmert-Str. 2-3, 14482 Potsdam, Germany

[14 June 2021]: notice amended to state that the funding information has been corrected to remove a duplicate reference to two funding organizations introduced in error during the initial correction.

In the originally published version of this manuscript, requested author amendments to the Funding section were inadvertently omitted prior to publishing. The section should read: ``German Federal Ministry of Research and Education (01ZZ1802); Physician-Scientist Program of the University of Heidelberg, Faculty of Medicine, DKTK (German Cancer Consortium) School of Oncology and the Cancer Core Europe TRYTRAC program (to A.M.); MTB-Report project (VolkswagenStiftung ZN3424) (to J.H.).'' instead of ``German Federal Ministry of Research and Education (01ZZ1802); University of Heidelberg, Faculty of Medicine (to A.M.); Volkswagen Foundation (to J.H.).'' This has now been corrected.

