# Supplementary material for: Knowledge bases and software support for variant interpretation in precision oncology
Source: Brief Bioinform. 2021 May 10;22(6):bbab134. doi: 10.1093/bib/bbab134 (PMC8574624; doi:10.1093/bib/bbab134)
Supplement: Suppl4_Prisma_Flow_Diagram_bbab134 [file suppl4_prisma_flow_diagram_bbab134.pdf]

PRISMA Flow Diagram for
Identifying Cancer Variant Annotation Tools

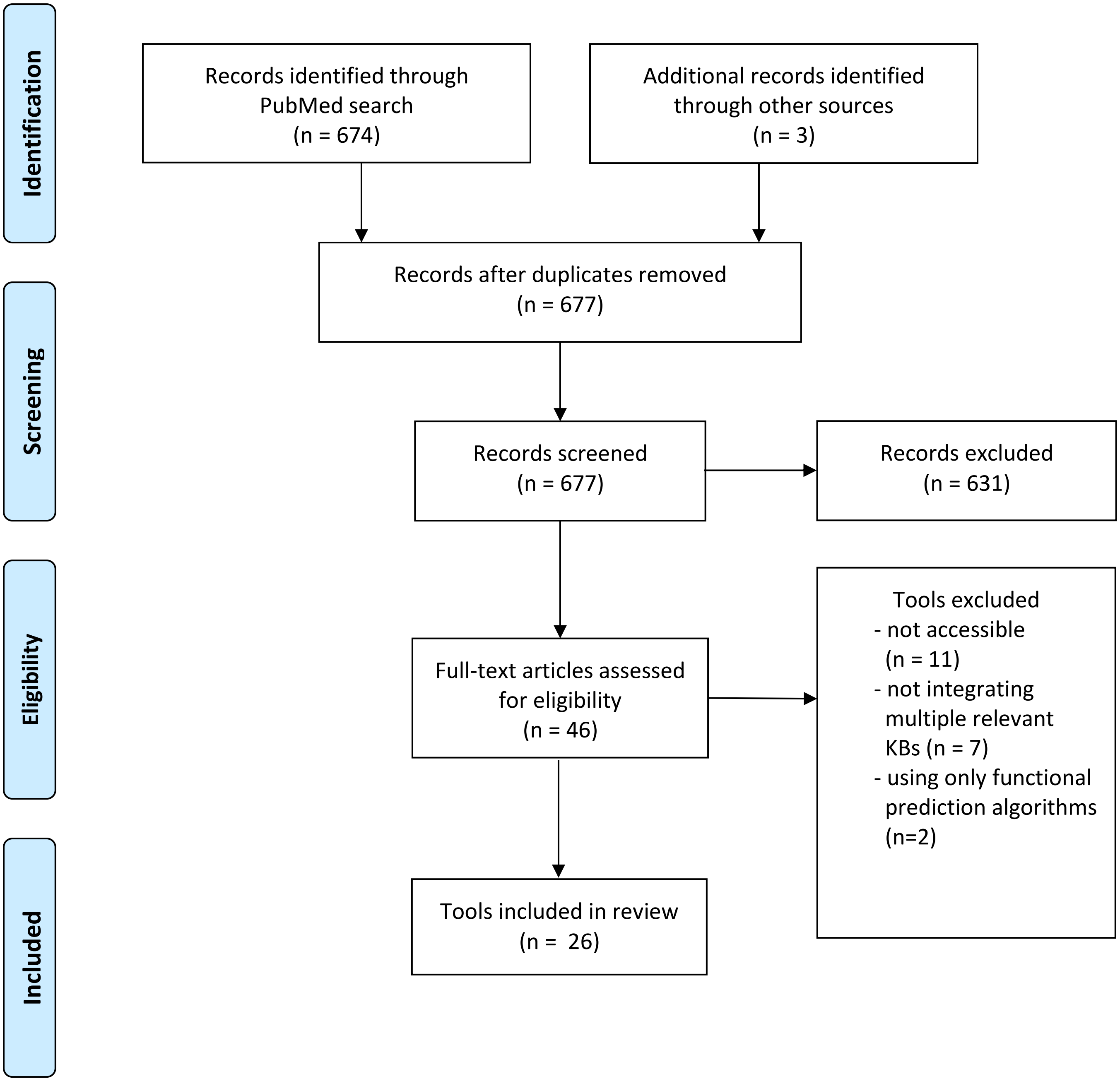

From: Moher D, Liberati A, Tetzlaff J, Altman DG, The PRISMA Group (2009). *P*referred *R*eporting *I*tems for *S*ystematic Reviews and *M*eta-*A*nalyses: The PRISMA Statement. PLoS Med 6(7): e1000097. doi:10.1371/journal.pmed1000097

For more information, visit www.prisma-statement.org.

Search String

PubMed:

(
("cancer" OR "oncology" OR "tumor" OR "tumour")
AND ("variant" OR "variants")
AND ("interpretation" OR "annotation" OR "analysis" OR "analyses" OR "reporting" OR "information")
AND ("database" OR "databases" OR "knowledge base" OR "knowledge bases" OR "knowledgebase" OR "knowledgebases" OR "integration" OR "integrated" OR "harmonized" OR "public knowledge")
AND ("software" OR "tool" OR "Information System" OR "GitHub" OR "application" OR "knowledge base" OR "knowledge bases" OR "knowledgebase" OR "knowledgebases"))
AND
(("1970/01/01"[Date - Entry] : "2020/12/09"[Date - Entry])
)
